# Islet transplantation into brown adipose tissue can delay immune rejection

**DOI:** 10.1172/jci.insight.152800

**Published:** 2022-02-22

**Authors:** Jessica D. Kepple, Jessie M. Barra, Martin E. Young, Chad S. Hunter, Hubert M. Tse

**Affiliations:** 1 Comprehensive Diabetes Center,; 2 Department of Medicine, Division of Endocrinology, Diabetes and Metabolism,; 3 Department of Microbiology, and; 4 Division of Cardiovascular Diseases, University of Alabama at Birmingham, Birmingham, Alabama, USA.

**Keywords:** Endocrinology, Transplantation, Autoimmune diseases, Glucose metabolism, Islet cells

## Abstract

Type 1 diabetes is an autoimmune disease characterized by insulin-producing β cell destruction. Although islet transplantation restores euglycemia and improves patient outcomes, an ideal transplant site remains elusive. Brown adipose tissue (BAT) has a highly vascularized and antiinflammatory microenvironment. Because these tissue features can promote islet graft survival, we hypothesized that islets transplanted into BAT will maintain islet graft and BAT function while delaying immune-mediated rejection. We transplanted syngeneic and allogeneic islets into BAT or under the kidney capsule of streptozotocin-induced diabetic NOD.*Rag* and NOD mice to investigate islet graft function, BAT function, metabolism, and immune-mediated rejection. Islet grafts within BAT restored euglycemia similarly to kidney capsule controls. Islets transplanted in BAT maintained expression of islet hormones and transcription factors and were vascularized. Compared with those in kidney capsule and euglycemic mock-surgery controls, no differences in glucose or insulin tolerance, thermogenic regulation, or energy expenditure were observed with islet grafts in BAT. Immune profiling of BAT revealed enriched antiinflammatory macrophages and T cells. Compared with the kidney capsule control, there were significant delays in autoimmune and allograft rejection of islets transplanted in BAT, possibly due to increased antiinflammatory immune populations. Our data support BAT as an alternative islet transplant site that may improve graft survival.

## Graphical abstract

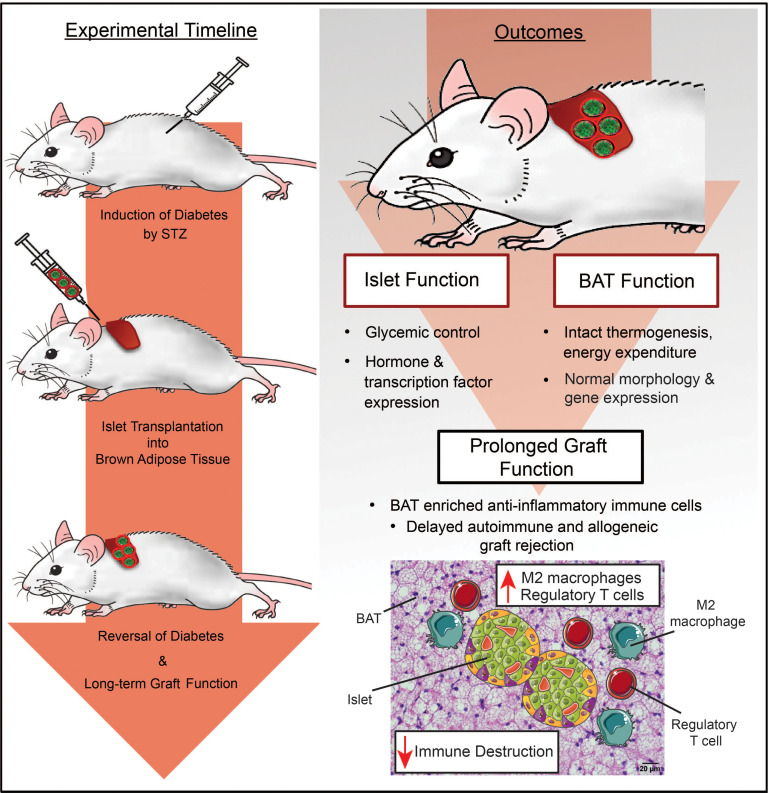

## Introduction

Type 1 diabetes (T1D) is characterized by the immune-mediated destruction of insulin-producing β cells. The subsequent lack of endogenous insulin results in severe glycemic fluctuations and risks for complications like cardiovascular disease, renal failure, and retinopathy (1). Pancreatic islet transplantation can restore glucose homeostasis and prevent hypoglycemic unawareness events, but long-term viability and function of the islet graft remain challenges. Currently, clinical islet transplants are performed into the hepatic portal vein. However, many studies have demonstrated that instant blood-mediated inflammatory reactions, thrombosis, and hepatic ischemia can lead to a loss of up to 70% of the transplanted islet mass (2–4). These shortcomings have prompted a search for alternative transplant sites (5).

A site with potential to improve the outcomes of islet transplantation is brown adipose tissue (BAT). Unlike white adipose tissue (WAT), which largely functions to store and release energy, BAT maintains thermogenesis by converting energy into heat (6). Rodents have a large, interscapular BAT deposit located superficially below the skin, and human imaging studies unequivocally demonstrated the presence of functional BAT in the interscapular region during infancy through childhood (>10 years, at least) (7–9). Adult humans have subcutaneous supraclavicular depots and deeply situated spinal, renal, and aortic BAT depots (7, 10, 11). BAT regulates metabolic homeostasis in part through the ability to sense and respond to changes in glucose and insulin levels (12). In preclinical models, transplantation of brown adipocyte progenitors into mice improved glycemic control and insulin sensitivity, highlighting the protective role of BAT against metabolic dysregulation in obesity and type 2 diabetes (12–14).

In the context of T1D, the BAT microenvironment may be beneficial for islet transplantation. BAT is densely vascularized and innervated, which may support islet graft survival by providing a nutrient-rich milieu (15, 16). BAT also contains niches of perivascular mesenchymal stem cells (MSCs), which are beneficial for islet transplant survival (6, 17–19). Additionally, unlike other adipose depots with highly inflammatory microenvironments, BAT displays an overall antiinflammatory phenotype (20). Compared with WAT, BAT is enriched for alternatively activated M2 macrophages and immunosuppressive Tregs important in regulating BAT-energy homeostasis (21, 22). The presence of these immune populations within BAT may be beneficial for islet engraftment by dampening proinflammatory immune responses after transplantation. Recently, it was demonstrated that islets transplanted into the BAT of streptozotocin (STZ)-treated C57BL/6 mice can restore euglycemia (23). Although this work established that islets can be transplanted into BAT, the effects on BAT function, immune composition, and immunoprotection after transplantation have yet to be explored. Here, we tested the hypothesis that islet transplantation into BAT of autoimmune-prone T1D NOD mice will promote normal islet graft and BAT function while delaying immune-mediated rejection. To our knowledge, this study is the first to demonstrate the protective advantage of BAT in maintaining both islet and BAT function and delaying islet allograft rejection in the absence of immunosuppression.

## Results

### Islet transplantation into BAT restores euglycemia and maintains glucose and insulin tolerance.

**Figure 1:**
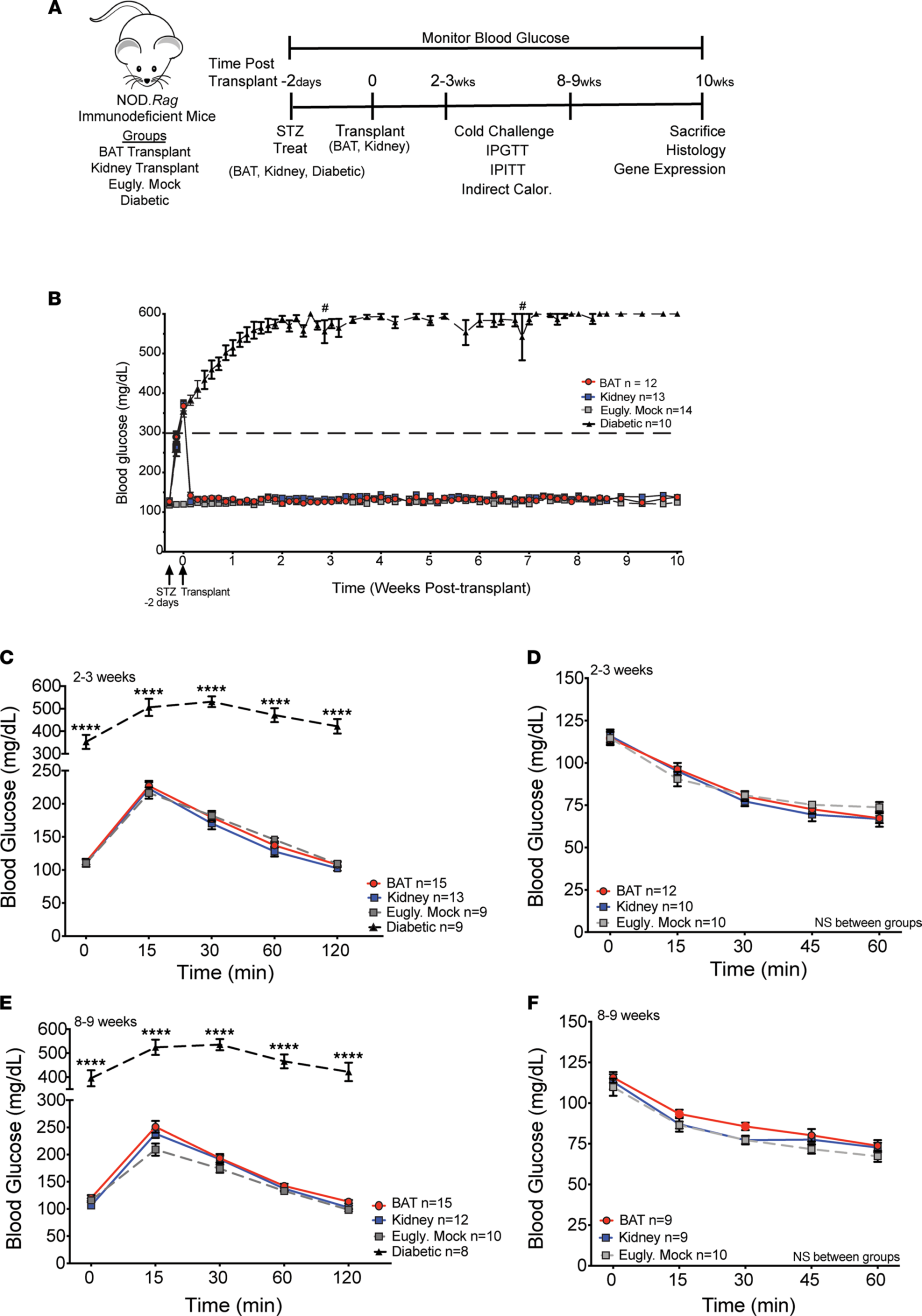
Islet transplantation into BAT restores euglycemia and maintains glucose and insulin tolerance. (**A**) Schematic highlighting the treatments and experimental timeline. (**B**) Daily ad libitum blood glucose levels over a span of 10 weeks (*n* = 10–14). (**C**) Glucose tolerance test conducted at 2–3 weeks after transplantation (*n* = 9–15). (**D**) Insulin tolerance test at 2–3 weeks after transplantation (*n* = 10–12). (**E**) Glucose tolerance test at 8–9 weeks after transplantation (*n* = 8–15). (**F**) Insulin tolerance test at 8–9 weeks after transplantation (*n* = 9–10). Analyzed by 2-way ANOVA with multiple comparisons and Tukey post hoc test. Data represents 4 independent experiments. Error bars are ± SEM. ****P < 0.0001. Calor, calorimetry; Eugly. mock, euglycemic mock control; IGITT, i.p. insulin tolerance testing; IGPTT, i.p. glucose tolerance testing; Treat, treatment.

To evaluate the feasibility of BAT as a transplant site in a diabetic mouse model, we first performed syngeneic transplants of 250 NOD.*Rag* islets into the BAT (denoted BAT group) of STZ-treated NOD.*Rag* mice (Figure 1A). As a comparator, islets were transplanted under the kidney capsule (denoted kidney group), a site widely used for islet transplantation in mice (24). STZ-treated, nontransplanted NOD.*Rag* diabetic controls and euglycemic mock-surgery controls were also included. To assess the ability of transplanted islets to restore euglycemia, we measured daily ad libitum blood glucose levels for 10 weeks after transplantation (Figure 1B). Following STZ treatment, all mice became hyperglycemic (blood glucose level ≥ 300 mg/dL). Within 48 hours after islet transplantation, BAT and kidney groups returned to euglycemia, similar to the euglycemic mock controls (Figure 1B). To determine potential differences in glucose and insulin tolerance between the BAT and kidney groups, we conducted i.p. glucose tolerance testing and i.p. insulin tolerance testing at 2 to 3 weeks (Figure 1, C and D) and 8 to 9 weeks (Figure 1, E and F) after transplantation. Compared with the euglycemic controls, there was no difference in glucose or insulin tolerance in the kidney and BAT groups. Overall, islet transplantation into BAT yielded restoration of euglycemia and maintenance of glucose and insulin tolerance comparable with islets engrafted under the kidney capsule.

### Islet grafts recovered from BAT express islet hormones and transcription factors.

**Figure 2:**
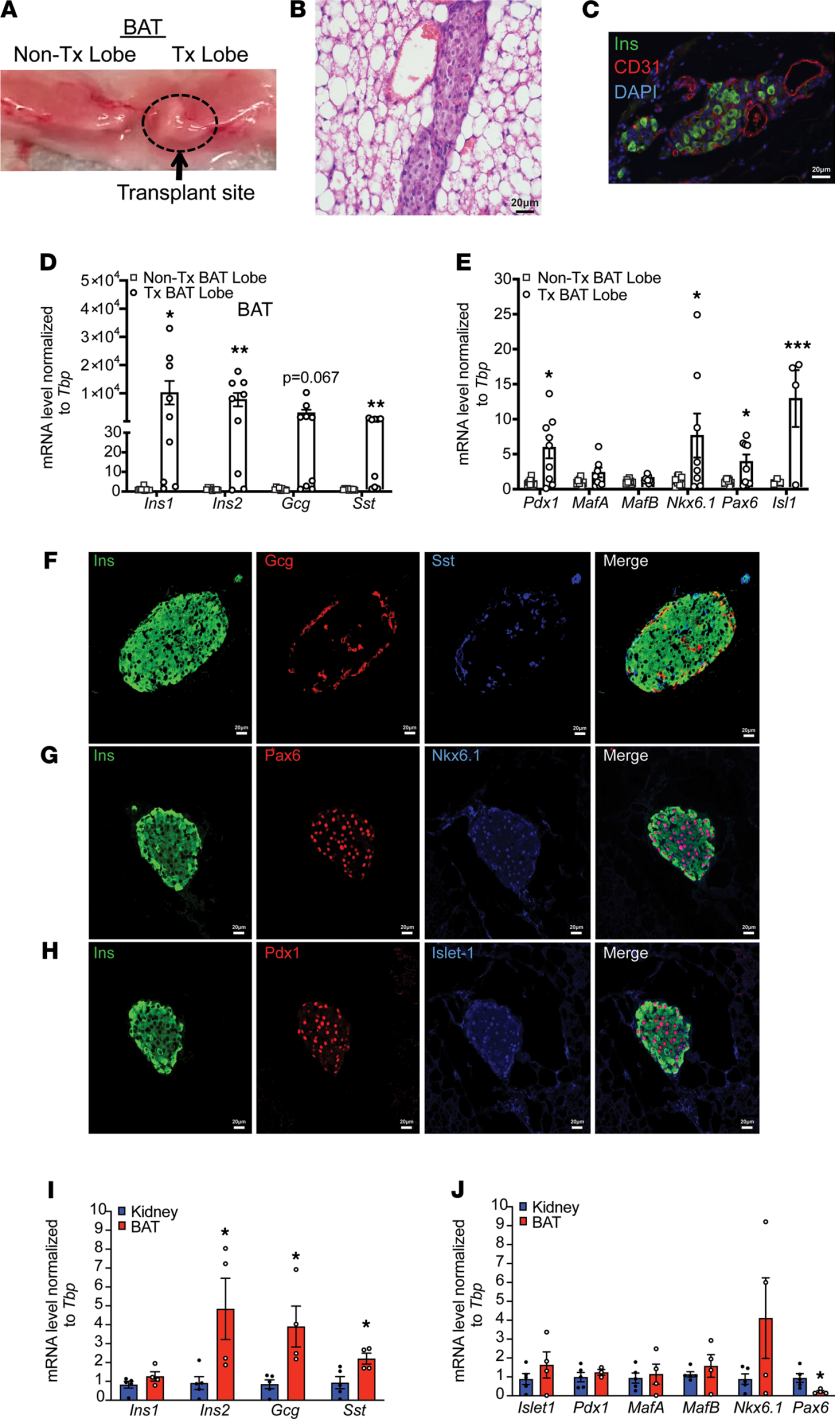
Islet grafts recovered from BAT express islet hormones and TFs. (**A**) Gross BAT morphology from BAT-engrafted group. Outline and arrow mark the inflated right BAT lobe where the islets were engrafted (*n* = 6). (**B**) H&E staining of engrafted BAT (*n* = 4). (**C**) Immunofluorescence of BAT group showing insulin (green), CD31 (red), and DAPI (blue) (*n* = 4). (**D** and **E**) qRT-PCR analyses between transplanted (Tx) and nontransplanted (non-Tx) BAT lobes for mRNA encoding islet hormones and critical TFs, respectively (*n* = 8–9). (**F**) Immunofluorescence for insulin (green), glucagon (red), and somatostatin (blue) and (**G** and **H**) islet TFs Pax6 (red), Nkx6.1 (blue), Pdx1 (red), and Islet-1 (blue) costained with insulin (green) (*n* = 3–4). Gene expression analysis for (**I**) islet hormones and (**J**) TFs from BAT and kidney islet grafts (*n* = 4–5). Analyzed with unpaired Student’s *t* tests. All histological images are ×40 magnification. Data represent at least 3 independent experiments. Error bars are ± SEM. *P < 0.05; **P < 0.01; ***P < 0.001.

To assess whether islets transplanted into BAT maintained expression of hormones and maturation markers, we performed histology and quantitative real-time PCR (qRT-PCR) on islet-engrafted BAT lobes at 10 weeks after transplantation. Gross inspection of the BAT depot revealed an inflated right BAT lobe indicative of the engraftment site (Figure 2A). Upon removal of the BAT lobes, we identified islets within the BAT via H&E staining (Figure 2B). Immunofluorescence demonstrated the presence of CD31^+^ vasculature adjacent to the islets (Figure 2C), suggesting that transplanted islets were revascularized in the BAT. Next, we assessed mRNA encoding islet hormones and transcription factors (TFs) required for islet function. Insulin (*Ins1*, *Ins2*), glucagon (*Gcg*), and somatostatin (*Sst*) mRNA levels were elevated in the islet-transplanted BAT lobe, as compared with the nontransplanted lobe (Figure 2D). Additionally, accumulation of critical TF-encoding *Pdx1*, *Pax6*, *Islet-1* (*Isl1*), and *Nkx6.1* mRNA was significantly enriched in the islet-transplanted BAT lobe corresponding to samples with elevated hormone mRNA (Figure 2E). *MafA* and *MafB* were not enriched in the islet-transplanted lobe, possibly due to endogenous expression of these factors in resident adipose cells, including macrophages and preadipocytes (25, 26) (Figure 2E). For islet hormone and TF expression, 3 of 9 samples did not have islet mRNA amounts significantly greater than in the nontransplanted BAT lobe, despite being euglycemic at the time of tissue collection. This is likely due to incomplete extraction of the engrafted site and loss of the islet tissue. Immunofluorescence of islets engrafted in BAT supported that insulin, glucagon, and somatostatin islet hormone expression was maintained (Figure 2F). These islets also expressed the β cell–enriched TFs Pdx1 and Nkx6.1, as well as the pan-islet TFs Pax6 and Islet-1 (Figure 2, G and H). The presence of these markers supports that islet transplanted into BAT maintained functional identity in vivo (27, 28). When compared with islets transplanted under the kidney capsule, BAT demonstrated an enrichment of mRNA encoding islet hormones but not TFs (Figure 2, I and J). At the protein level, the kidney group maintained similar islet hormone and TF expression (Supplemental Figure 2; supplemental material available online with this article; https://doi.org/10.1172/jci.insight.152800DS1).

### Islet engraftment into BAT does not affect energy expenditure and thermogenesis.

**Figure 3:**
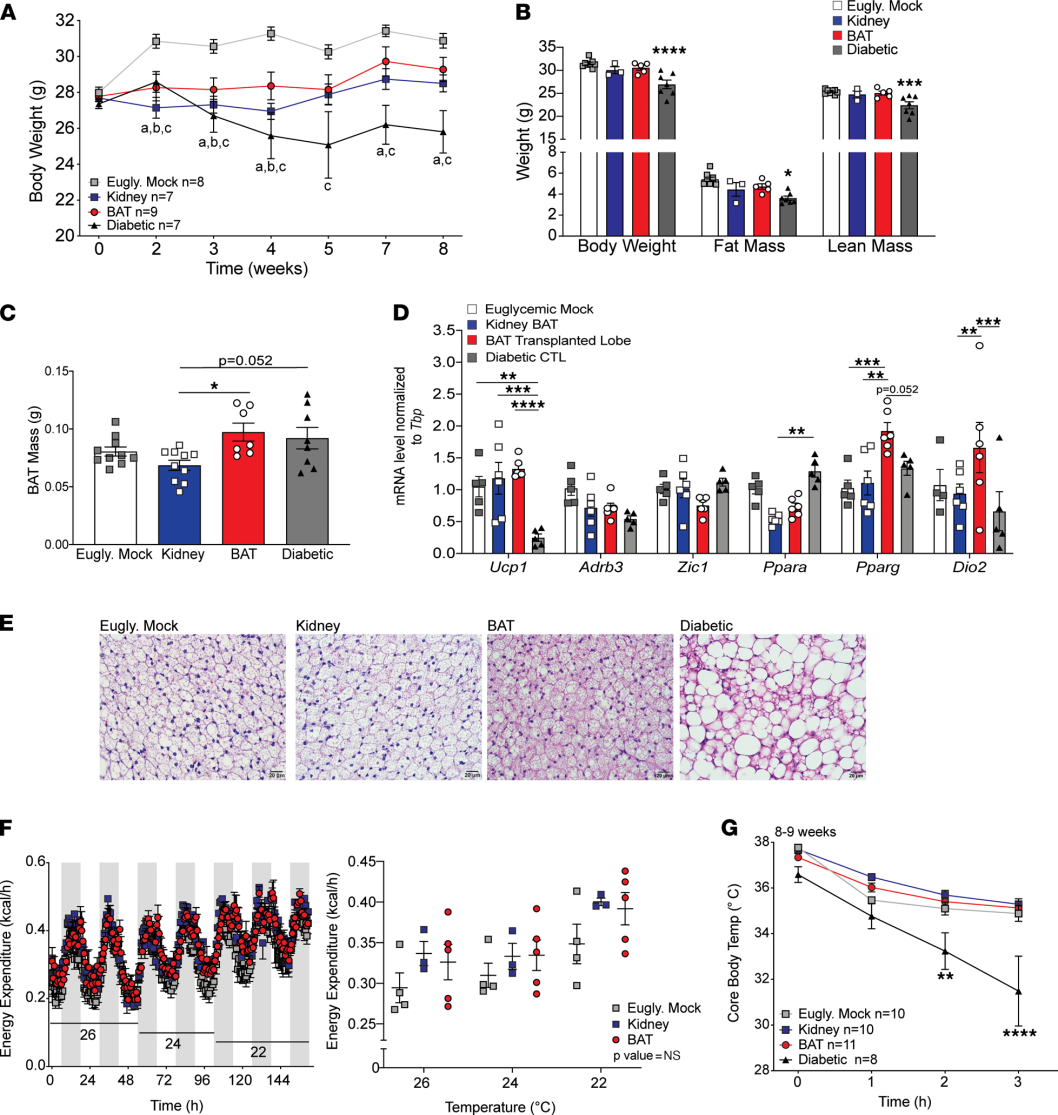
Islet transplantation into BAT does not affect energy expenditure and thermogenesis. (**A**) Assessment of BW during the transplant experiment. (**B**) Body composition, including lean and fat mass, was assessed for all groups via quantitative magnetic resonance; BAT (*n* = 5), kidney control (*n* = 3), euglycemic mock control (Eugly. mock; *n* = 8), diabetic control (*n* = 7). (**C**) BAT mass from all groups: BAT (*n* = 7), kidney (*n* = 10), Eugly. mock (*n* = 10), and diabetic (*n* = 8). (**D**) qRT-PCR analysis of gene expression from BAT in each group assessing BAT markers and TFs (*n* = 4–6). (**E**) H&E staining of BAT from all groups (*n* = 3) at ×40 magnification. (**F**) Indirect calorimetry analysis of the kidney (*n* = 3), BAT (*n* = 5), and Eugly. mock (*n* = 4) control groups. Energy expenditure measured at 26°C, 24°C, and 22°C in chow-fed mice. (**G**) Cold challenge conducted for 3 hours at 4°C at 8–9 weeks (*n* = 9–10). Body composition and energy expenditure analyzed by 1-way or 2-way ANOVA with Tukey post hoc test, compared with Eugly. mock. For BW analysis, the letters represent significant differences between the Eugly. mock and (a) kidney, (b) BAT, and (c) diabetic control. Data represent at least 3 independent experiments. Error bars are ± SEM. *P < 0.05; **P < 0.01; ***P < 0.001; ****P < 0.0001.

Given the contribution of BAT to maintaining whole-body energy balance (29), we next investigated whether islet transplantation negatively affected BAT function. We measured BW over time after islet engraftment (Figure 3A). Compared with euglycemic mock controls, all STZ-treated groups had slightly lower BWs at 2 weeks after STZ treatment. By 8 weeks after transplantation, there was no significant difference in BW among the BAT, kidney, and euglycemic mock groups. Assessment of body composition at 9 weeks after transplantation revealed no differences among the BAT, kidney, and euglycemic mock groups (Figure 3B). Measurements of BAT mass, taken from extracted and trimmed BAT, revealed no difference at 10 weeks after transplantation, when comparing BAT and kidney with euglycemic mock controls (Figure 3C). Interestingly, the diabetic group had reductions in BW, body composition, and fat mass with no difference in BAT mass, when compared with the euglycemic mock controls (Figure 3, A–C).

We next performed qRT-PCR on BAT from all groups to assess mRNA associated with BAT identity and function (Figure 3D). When compared with euglycemic mock controls, the BAT engraftment group had no changes in BAT-specific mRNA encoding *Adrb3, Zic1*, and the critical, thermogenic, uncoupled protein *Ucp1*. Compared with controls, islet-engrafted BAT was enriched for *Pparg* and *Dio2* mRNA, encoding factors involved in adipogenesis and thermogenesis. This enrichment may be due to an effect of localized insulin secreted from the islet grafts, affecting adipogenesis. The diabetic group had significant reductions in mRNA encoding *Ucp1* and *Dio2*.

Next, we conducted H&E staining to assess potential changes in BAT morphology between groups. Unlike the diabetic group, which had more unilocular adipocytes indicative of altered BAT function, each transplant group had typical multilocular BAT morphology (Figure 3E). We also evaluated whether islet transplantation affected the ability of BAT to modulate whole-body energy expenditure (EE). To test this, we conducted indirect calorimetry at 3 temperatures (26°C, 24°C, 22°C) to assess temperature-dependent impacts on EE (Figure 3F). All groups followed typical diurnal patterns of EE, with peaks during the dark/active phases and troughs during the light/inactive phases. Additionally, EE increased as temperature was reduced, consistent with elevated thermogenic activity. Compared with euglycemic mock controls, there was no significant difference in EE at any temperature for the kidney or BAT groups. Additional parameters were assessed during the indirect calorimetry, including respiratory quotient, food intake, locomotion, and weekly BWs, all of which were unchanged among groups (Supplemental Figure 3, A–D).

Finally, to test whether islet transplantation into BAT alters thermogenesis, we conducted a cold challenge to measure changes in core body temperature during acute cold exposure. At both 2 to 3 weeks and 8 to 9 weeks after transplantation (Supplemental Figure 3E and Figure 3G), apart from the diabetic controls that had significant reductions in body temperature over time, all groups were able to defend body temperature. This supports that islet transplantation does not affect BAT function and may also protect against the BAT dysfunction observed in the hyperglycemic diabetic group.

### BAT displays an enhanced antiinflammatory immune profile compared with kidney.

Since transplanted islets into BAT are functional, we wanted to assess the ability of engrafted islets to withstand immune rejection, compared with islets transplanted under the kidney capsule. Relative to other adipose depots, BAT displays an inherent antiinflammatory immune profile comprising alternatively activated M2 macrophages and Tregs (20, 30), whereas the kidney has an inflammatory immune profile containing IFN-γ^+^ CD4^+^ T cells and a large number of neutrophils (31, 32). However, to our knowledge, immunophenotyping and comparative analysis of these two sites for the frequency and number of pro- and antiinflammatory immune cells have not been conducted. Therefore, we performed flow cytometry to investigate the immune composition of naive BAT versus kidney from NOD mice.

**Figure 4:**
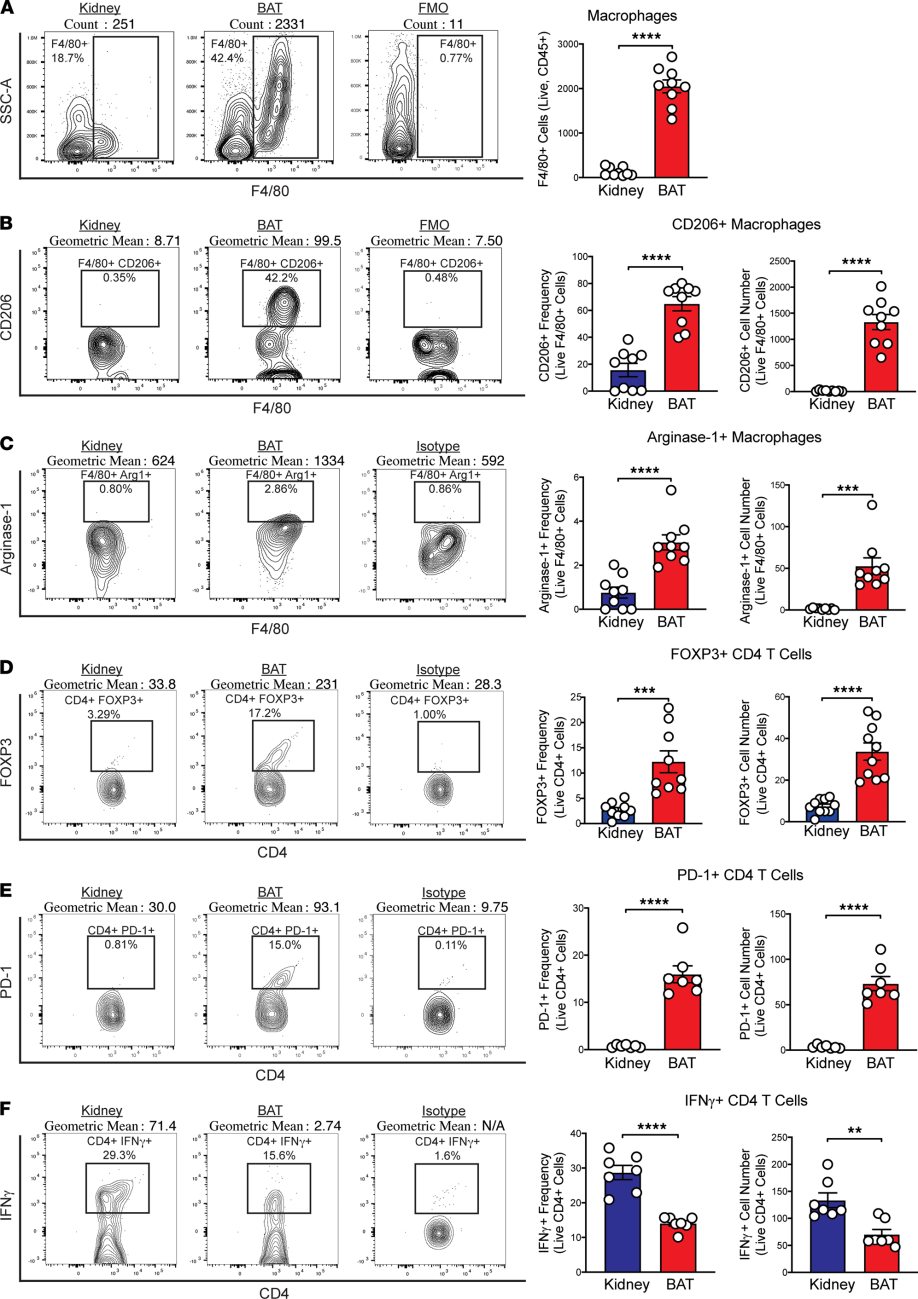
BAT displays an enhanced antiinflammatory immune profile compared with kidney. (**A–F**) Flow cytometry analysis of BAT and kidney single-cell suspensions from NOD mice for (**A**) number of F4/80^+^ macrophages (*n* = 7–9), frequency and number of (**B**) CD206^+^ F4/80^+^ macrophages (*n* = 7–9), (**C**) arginase-1^+^ F4/80^+^ macrophages (*n* = 7–9), (**D**) FOXP3^+^ CD4^+^ T cells (*n* = 7–9), (**E**) PD-1^+^ CD4^+^ T cells (*n* = 7–9), and (**F**) IFN-γ^+^ CD4^+^ T cells (*n* = 7–9). Analyzed with unpaired Student’s *t* test. Data represent 3 independent experiments. Error bars are ± SD. **P < 0.01; ***P < 0.001; ****P < 0.0001. FMO, fluorescence minus one control; Isotype, IgG isotype control.

Compared with kidney, BAT demonstrated a significant increase in the number of F4/80^+^ macrophages (Figure 4A) and a significantly increased frequency and number of CD206^+^ and arginase-1^+^ alternatively activated M2 macrophages (Figure 4, B and C). BAT was also enriched for CD4^+^ Tregs (30). BAT also had significantly higher frequencies and numbers of CD4^+^ T cells expressing the key regulatory TF FOXP3 (Figure 4D) and the immunoregulatory receptor programmed cell death-1 (PD-1) (Figure 4E) involved in the suppression of effector T cell responses (33). BAT also displayed a significant reduction in the frequency and number of proinflammatory IFN-γ^+^ CD4^+^ T cells (Figure 4F). These results suggest that the native antiinflammatory immune environment of BAT may be beneficial for islet survival after transplantation, compared with kidney capsule.

### Islets transplanted into BAT delay autoimmune-mediated graft rejection.

**Figure 5:**
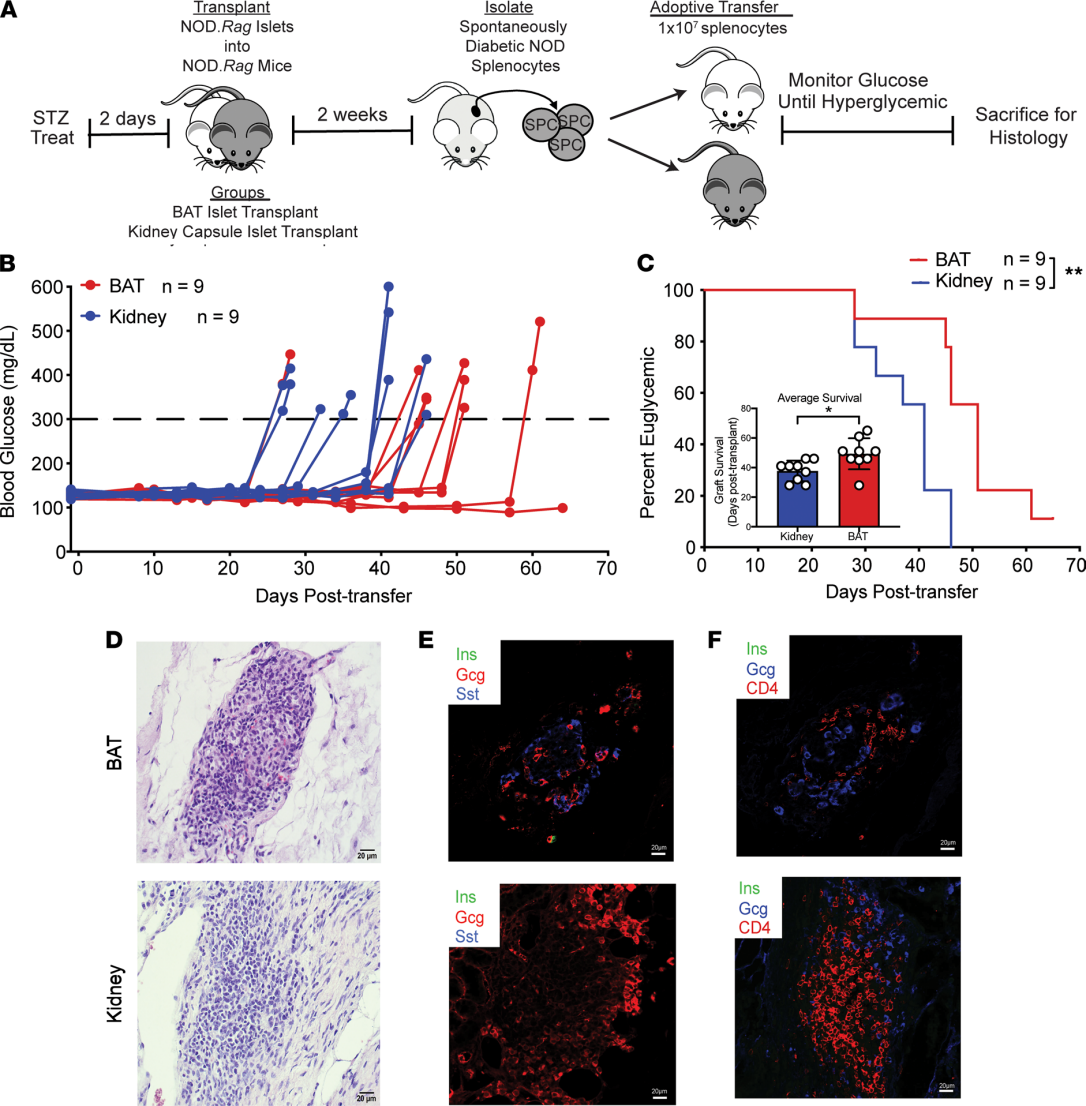
Islets transplanted into BAT delay autoimmune-mediated graft rejection. (**A**) Schematic of adoptive transfer experimental design. (**B**) Ad libitum blood glucose values over time after adoptive transfer of 1 × 10^7^ spontaneously diabetic NOD splenocytes (*n* = 9). (**C**) Kaplan-Meier log-rank test for percentage of adoptive transfer recipients maintaining islet graft function, based on blood glucose readings (*n* = 9). Inset displaying individual recipient graft survival analyzed via Student’s *t* test. (**D**) H&E staining of islet grafts from BAT (day 51 after Tx) and kidney capsule (day 41 after Tx) (*n* = 3–4). (**E** and **F**) Immunofluorescence of insulin (green), glucagon (red or blue), somatostatin (blue), and CD4 T cells (red) (*n* = 3–4). All histology images are ×40 magnification. Data represent 3 independent experiments. Error bars are ± SD. *P < 0.05; **P < 0.01. Treat, treatment; Tx, transplantation.

Because naive BAT displayed enhanced antiinflammatory immune profiles compared with kidney, we sought to determine whether the BAT microenvironment could elicit a delay in autoimmune-mediated islet graft rejection compared with the kidney capsule site. To test this, we performed an adoptive transfer of splenocytes from diabetic NOD mice into euglycemic immune deficient NOD.*Rag* recipients with islets transplanted into BAT or under the kidney capsule (Figure 5A). Blood glucose level was measured over time to assess graft rejection. All groups were euglycemic at the time of transfer, with some recipients becoming hyperglycemic at 25 days after transfer (Figure 5B). Recipients with islets transplanted into the BAT maintained euglycemia significantly longer (1.3-fold) than recipients with islets transplanted under the kidney capsule (Figure 5C).

To confirm that islet grafts were rejected because of transferred diabetogenic splenocytes, we performed H&E staining and immunofluorescence. We identified islets within the respective sites with evidence of insulitis surrounding the islet grafts (Figure 5D). Immunofluorescence revealed loss of insulin expression within islets costained with glucagon and somatostatin in both groups (Figure 5E). Loss of insulin positivity was consistent with hyperglycemia of recipients following adoptive transfer with diabetogenic NOD splenocytes. Additionally, both groups displayed CD4^+^ T cell insulitis around the islet grafts, supporting that diabetes was due to islet graft destruction by adoptively transferred T cells (Figure 5F).

### Islets transplanted into BAT delay allograft rejection.

**Figure 6:**
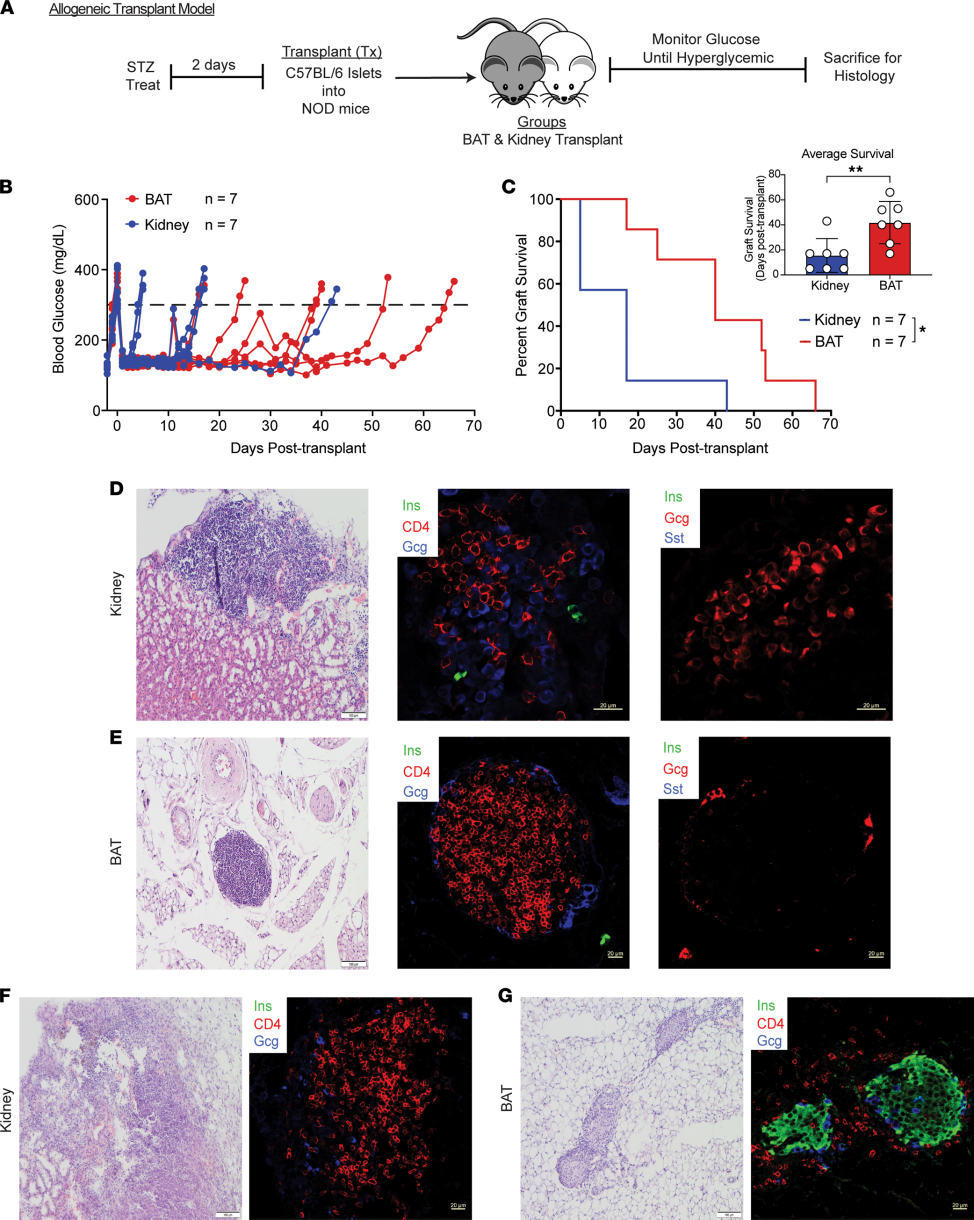
Islets transplanted into BAT delay allograft rejection. (**A**) Schematic of allogeneic islet transplant experimental design. (**B**) Ad libitum daily blood glucose values after allogeneic islet transplantation into NOD recipients (*n* = 7). (**C**) Kaplan-Meier log-rank test for percentage of allograft recipients maintaining islet graft function, based on blood glucose readings (*n* = 7). Inset displaying individual recipient graft survival analyzed via Student’s *t* test. (**D** and **E**) Staining of islet grafts from BAT (day 66 after Tx) and kidney capsule (day 5 after Tx) via H&E and immunofluorescence staining of insulin (green), glucagon (red or blue), somatostatin (blue), and CD4 T cells (red) (*n* = 3–4). (**F** and **G**) Staining of islet grafts from BAT and kidney capsule (both day 10 after Tx) via H&E and immunofluorescence staining of insulin (green), glucagon (blue), and CD4 T cells (red) (*n* = 3–4). All histology images are ×40 magnification. Data represent 3 independent experiments. Error bars are ± SD. *P < 0.05; **P < 0.01. Tx, transplantation.

To assess the efficacy of the BAT site to delay islet graft rejection in a clinically relevant transplant model involving autoimmune and allogeneic immune responses, we next transplanted C57BL/6 islets into the BAT or under the kidney capsule of STZ-treated NOD mice in the absence of global immunosuppression (Figure 6A). All islet allograft recipients returned to euglycemia within 48 hours after transplantation (Figure 6B), but islets transplanted into BAT maintained euglycemia significantly longer (2.7-fold) than those engrafted under the kidney capsule in the absence of systemic immunosuppression (Figure 6, B and C). H&E and immunofluorescence staining performed on failed islet allografts collected from hyperglycemic mice at 5 days after transplant for the kidney group and 66 days after transplant for BAT confirmed allograft rejection, as shown by insulitis, increased number of CD4^+^ T cells, and loss of insulin expression (Figure 6, D and E). Additionally, histological analysis of euglycemic islet-engrafted mice at 10 days after transplant revealed an increase in insulitis consisting of CD4^+^ T cells, CD8^+^ T cells, and loss of insulin expression in the kidney group, as compared with the BAT group (Figure 6, F and G; and Supplemental Figure 4). These data provide evidence that BAT as an engraftment site can significantly delay autoimmune and allogeneic islet destruction, compared with islets transplanted under the kidney capsule.

### BAT maintains an antiinflammatory immune profile after allogeneic islet transplantation.

**Figure 7:**
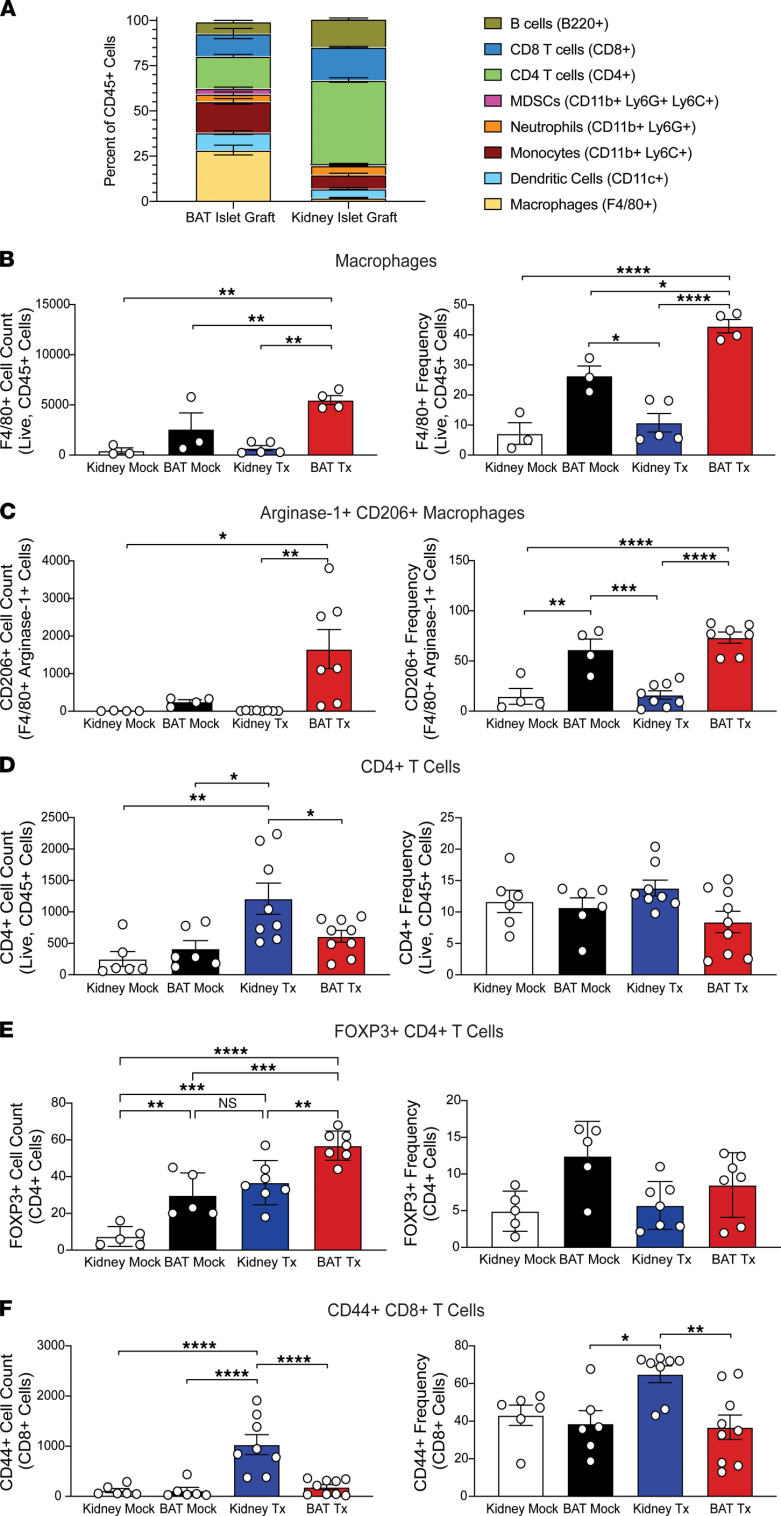
BAT maintains an antiinflammatory immune profile after allogeneic islet transplantation. (**A–F**) Flow cytometry analysis of mock surgery controls or allogeneic C57BL/6 islet grafts transplanted in BAT or under the kidney capsule of NOD mice on day 14 (**B** and **C**) and day 10 (**A** and **D–F**) after transplantation. (**A**) Composition of CD45^+^ leukocytes within BAT or kidney capsule islet grafts (*n* = 4). Number and frequency of (**B**) F4/80^+^ macrophages, (**C**) CD206^+^ arginase-1+ F4/80^+^ macrophages, (**D**) CD4^+^ T cells, (**E**) FOXP3^+^ CD4 T cells, and (**F**) CD44^+^ CD8^+^ T cells (*n* = 3–9 for **B–F**). Data were analyzed by 1-way ANOVA with multiple comparison and Tukey post hoc test. Data represent 3 independent experiments. Error bars are ± SD. *P < 0.05; **P < 0.01; ***P < 0.001; ****P < 0.0001. Mock, mock control; Tx, transplantation.

Having characterized an enrichment of antiinflammatory immune cells in naive BAT versus kidney (Figure 4), we wanted to determine if these populations were maintained after islet transplantation. To compare immune populations during graft rejection, we performed flow cytometry analysis of C57BL/6 islet allografts from BAT or kidney capsule at 10 and 14 days after transplantation into NOD mice. As comparators, we also included BAT and kidney mock-surgery controls. Assessment of total CD45^+^ leukocytes for B220^+^ B cells, CD8^+^ T cells, CD4^+^ T cells, CD11b^+^ Ly6G^+^ Ly6C^+^ myeloid-derived suppressor cells (MDSCs), CD11b^+^ Ly6G^+^ Ly6C^–^ neutrophils, CD11b^+^ Ly6C^+^ Ly6G^–^ monocytes, CD11c^+^ DCs, and F4/80^+^ macrophages within islet grafts revealed notable differences in immune composition between tissues after transplantation (Figure 7A). BAT displayed an enrichment in the frequency of macrophages and reductions in numbers of CD4^+^ T cells, as we demonstrated in our previous analysis of naive tissues (Figure 4). We also identified a small frequency of MDSCs within BAT islet grafts that was absent in islets transplanted under the kidney capsule, and a reduction in the frequency of B220^+^ B cells within BAT islet grafts, compared with kidney (Figure 7A).

Given the published literature identifying M2 macrophages and Tregs as key populations within BAT, we further analyzed these populations from our islet graft recipients. Assessment of macrophages demonstrated an enrichment in both number and frequency from islets engrafted into BAT, compared with mock-surgery controls and islets transplanted under the kidney capsule (Figure 7B). Islet allografts into BAT had significantly increased number and frequency of CD206^+^ arginase-1^+^ alternatively activated M2 macrophages when compared with the kidney groups (Figure 7C) and also had an increase in arginase-1^+^ DCs compared with all other groups (Supplemental Figure 5A).

Next, we investigated the T cell phenotype between groups and observed a significant decrease in CD4^+^ T cell numbers with islets transplanted into the BAT compared with islets engrafted into the kidney capsule (Figure 7D). Strikingly, compared with islets engrafted under the kidney capsule, allotransplanted islets into BAT had significantly more CD4^+^ T cells expressing FOXP3 (Figure 7E), a TF involved in Treg differentiation, and inhibitory receptor PD-1 (Supplemental Figure 5B). There was also a reduction in the number of activated CD44^+^ CD4^+^ T cells within BAT islet grafts compared with islets within the kidney capsule; however, the frequencies were unchanged (Supplemental Figure 5C). We also found a significant decrease in the number and frequency of total CD8^+^ T cells (Supplemental Figure 5D) and activated CD44^+^ CD8^+^ T cells (Figure 7F) in islets engrafted into BAT, compared with under the kidney capsule. Conversely, islets engrafted to BAT had a significant increase in CD8^+^ T cells expressing the immunomodulatory PD-1^+^ cell surface receptor (Supplemental Figure 5E). Overall, these data provide evidence that islets transplanted into BAT can elicit a delay in autoimmune and allograft rejection.

## Discussion

Islet transplantation is a promising treatment to restore glucose homeostasis in patients with T1D. Current clinical transplantation practices use the hepatic portal vein as the site for islet transplantation in humans. Despite short-term success, most transplant recipients do not remain insulin independent 5 years after transplantation, because of immunological and physiological damage to the islet graft (24). In mouse studies, the kidney capsule is the preferred site for islet transplantation, because of ease of surgical transplantation and graft retrievability (5, 24). Physiological differences between mouse and human kidneys, including relatively poor blood supply in humans, likely prevents clinical translation. Alternative sites have been proposed to address challenges with immunological rejection, survival of engrafted islets, and surgical accessibility (5). These sites include the spleen, bone marrow, and skeletal muscle, as well as various fat depots, including the peritoneum, omentum, and subcutaneous depots. Although a few of these sites have shown promise and progressed to clinical trials (5), the large number of islets required to restore euglycemia and long-term graft survival remains a challenge. In this preclinical mouse study, we demonstrated that islets transplanted into BAT restored euglycemia within 24 hours after transplantation, preserved islet and BAT function, and exhibited a delay in immune rejection, compared with islets transplanted under the kidney capsule.

BAT is densely vascularized and innervated and, unlike the portal vein, where high blood flow can damage engrafted islets, the dense peripheral vasculature in BAT should allow for proper oxygenation while limiting sheer stress to the islets. Additionally, BAT contains niches of perivascular MSCs, which promote the survival of engrafted islets (18, 34). Unlike other alternative sites proposed, BAT is a metabolic tissue with roles in regulating glucose homeostasis. Transplantation of brown adipocytes into diabetic mice can restore euglycemia and normalize glucose tolerance (12). Given the importance of BAT in maintaining metabolic homeostasis, our data demonstrated that islet transplantation into BAT does not compromise tissue function.

The STZ-treated diabetic control mice in this study revealed the treatment had negative impacts on body composition, impaired thermogenesis, altered BAT morphology, and reduced *Ucp1* mRNA levels. Restoration and maintenance of euglycemia upon islet transplantation prevented BAT dysfunction. Despite impacts of hyperglycemia on BAT function, there was no change in BAT mass within this diabetic control group. Gross assessment of the supraclavicular fat pad revealed retention of BAT with an overt loss of WAT (Supplemental Figure 6), suggesting chronic hyperglycemia affected adipose depots differently. However, our STZ-treated diabetic control group represented a much more severe phenotype compared with patients with T1D who receive insulin therapy, because these mice experience chronic untreated hyperglycemia. Assessment of glucose uptake via PET-CT imaging demonstrated metabolically active BAT in patients living with T1D for up to 16 years (35), suggesting that human BAT may maintain tissue function regardless of the level of glycemic control and insulin dependence.

BAT displays promising characteristics ideal for an islet transplant site in diabetic mouse models. Previous work demonstrated that syngeneic C57BL/6 islets transplanted into BAT could restore euglycemia (23). However, the impact of islet transplantation on the function of BAT, as well as the potential benefits of the antiinflammatory microenvironment of BAT, were not explored. The BAT microenvironment contains resident antiinflammatory immune cells that may benefit islet graft survival. In studies in which WAT and BAT immune populations were compared, researchers demonstrated that BAT has fewer macrophages and is less prone to inflammation, suggesting it may be more immunoprotective than other adipose depots (36, 37). Our immunophenotyping studies in BAT versus the kidney revealed an enrichment of alternatively activated M2 macrophages expressing CD206 and arginase-1, which can promote BAT thermogenic function (22, 38). We also observed an increase in FOXP3^+^ Tregs, which can suppress tissue inflammation, in part through M2 activation (30), and an increase in arginase-1^+^ DCs, indicative of a regulatory DC population (39). The presence of M2 macrophages, Tregs, and regulatory DCs could mitigate proinflammatory immune responses against engrafted islets upon transplantation (40, 41). We demonstrated that the number and frequency of proinflammatory IFN-γ^+^ CD4^+^ T cells in BAT were significantly reduced compared with kidney, further supporting BAT as an inherently antiinflammatory site for islet transplantation.

In support of BAT as an antiinflammatory transplant site, islet transplants into BAT had a significant delay in immune-mediated graft rejection after adoptive transfer with diabetic NOD splenocytes and in an allogeneic islet transplant model. Strikingly, the improved graft survival of islets allotransplanted in BAT was achieved in the absence of systemic immunosuppression. Immunophenotyping studies 10 to 14 days after allogeneic transplantation revealed that alternatively activated M2 macrophages were enriched in BAT, Treg populations were maintained, and there was a reduction of T cell infiltration into BAT, compared with islets transplanted under the kidney capsule. The role of antiinflammatory M2 macrophages and Tregs within BAT and whether they can confer immunoprotection have yet to be elucidated. More studies are required to determine if these immune subsets are responsible for the delay in islet graft survival, compared with kidney. Finally, novel strategies to specifically deplete M2 macrophages and Tregs in BAT are needed to demonstrate that these antiinflammatory immune cells can provide immunoprotection of transplanted islets.

Although not the focus of this study, we also observed a population of MDSCs present within islet grafts in BAT that were absent in islets transplanted under the kidney capsule. This subset of myeloid cells previously demonstrated protective effects when cotransplanted with allogeneic islets, by increasing Tregs (42), suggesting that MDSCs in BAT may also be contributing to the immunosuppressive environment in BAT and facilitating a delay in islet graft rejection. More studies are required to elucidate the role of this immune subset. Overall, these results highlight that the delay in islet graft rejection in BAT may be due, in part, to the maintenance of antiinflammatory immune cells. Future studies will define the tolerogenic potential of the BAT microenvironment. It is plausible that uncharacterized immune and/or nonimmune cells in the BAT or endogenous antiinflammatory molecules could play a role in maintaining an antiinflammatory microenvironment. By using single-cell RNA sequencing, immunophenotyping, and proteomics of BAT, we may identify novel pathways and/or cell types that can be exploited to enhance islet graft survival.

Given that BAT is highly vascularized and has endocrine function, transplanting into BAT may affect the survival of islet grafts independent of immune rejection. For example, the highly vascularized microenvironment of BAT may promote greater graft survival and function after transplantation, compared with the kidney capsule. Additionally, the minimum number of islets required to maintain euglycemia between transplant sites has yet to be defined, although a previous study demonstrated that euglycemia can be achieved by transplanting a marginal islet mass of 80 islets into BAT (23). If BAT can restore euglycemia with fewer islets compared with the kidney capsule or portal vein, this alternative transplant site may improve results from islet autotransplant in patients with pancreatitis (43–45), where lower islet numbers from the damaged pancreas may contribute to reduced transplant success. In addition, transplantation studies incorporating engraftment into BAT with other therapeutic strategies could further enhance islet graft survival. These approaches include induction of localized immunosuppression by encapsulating islets with antioxidant-containing nanothin coatings to decrease oxidative stress (46), incorporating dexamethasone-eluting micelles to suppress inflammatory responses (47) or by cotransplanting BAT-derived MSCs to further induce Treg differentiation (42). Additionally, BAT is a promising extrahepatic transplant site for other sources of insulin-producing β cells such as stem cell-derived β cells (48–51) or genetically modified xenogeneic porcine islets (52–57), both of which have demonstrated promise. However, the maximum possible islet number for a single BAT depot would have to be defined in both these preclinical models and also in human BAT to determine if the higher number of islet equivalents needed in these alternative approaches would be feasible.

Adipose tissue can also be used as a material to improve islet transplant efficacy. It contains a widely available source of MSCs that functions to promote tissue repair and regulate immune responses (58). Cotransplantation of islets with adipose-derived MSCs stimulates revascularization of the islet graft and reduces inflammatory immune responses (18, 34). Induction of angiogenesis in engrafted tissue is mediated by various MSC-secreted factors like VEGF, HGF, and TGF-β (19, 53). MSCs may also have immunomodulatory roles that enhance islet graft survival. MSC cotransplantation reduces proinflammatory cytokine secretion and CD4^+^ and CD8^+^ T cell trafficking while promoting antiinflammatory Treg responses (59). Additionally, MSCs directly promote islet graft survival by stimulating β cell proliferation and enhancing insulin secretion, possibly via the secretion of growth factors and IL-6 (60). Given that MSCs compose approximately 3% to 5% of the cells in adipose tissue, they represent a widely available and easily accessible cell type that can be used for stem cell therapies. Moving forward, a greater understanding of the cellular composition of BAT, as well as of specific regulatory pathways governing brown adipocyte proliferation and activity, will enhance the efficacy of these therapeutics.

Our data demonstrated that BAT is an efficacious site for islet transplantation that can promote islet graft survival and function and delay immune-mediated graft rejection without negatively affecting BAT function. Studies are needed to better understand the immune profile of BAT in healthy individuals and patients with T1D to help predict potential impacts on graft survival upon transplantation. It is unclear how human adipose tissue heterogeneity will affect the application of BAT as an engraftment site. Studies characterizing BAT mass and activity in patient populations should be conducted to better understand the application of BAT as a clinically relevant engraftment site. In preclinical models assessing long-term graft function, BAT may represent an untapped source of information regarding protective molecules or cell populations that can improve islet transplantation. A greater understanding of the specific protective mechanisms of BAT as a transplant site may help define therapeutic targets and improve the efficacy of clinical islet transplantation.

## Methods

### Mice.

Male NOD.*Rag*, NOD/LtJ, and C57BL/6 mice between 8 and 12 weeks of age were housed on a light/dark (12 h/12 h) cycle at 23°C with ad libitum access to standard laboratory chow and acidified water. To normalize the glycemic set point and synchronize diabetes of a large cohort of age-matched recipients, induction of diabetes was conducted as previously described with euglycemic NOD mice via STZ injection (190 mg/kg BW) (46, 61). Power analysis and sample size calculations were determined with online statistical resources (e.g., https://www.stat.ubc.ca/~rollin/stats/ssize/n2). For ≥80% power (α = 0.05) and an expected ≥20% mean difference in control and experimental groups, at least 6 to 15 animals per group were used.

### Islet transplantation.

Euglycemia was restored by transplanting 250 islets from NOD.*Rag* or C57BL/6 mice into diabetic NOD.*Rag* or NOD mice, respectively, under the kidney capsule as previously described (46). Islet transplantation into BAT was conducted via a scapular incision. The scapular white fat was cut and folded back to reveal the large scapular bifurcated BAT depot. Next, 250 islets were infused into the right lobe of the BAT through PE50 tubing with a micromanipulator syringe. The tubing was removed, and the fat was folded back. Finally, the skin sealed with 9 mm wound clips (Fine Science Tools).

### Histology.

The scapular fat pad was extracted, and the bifurcated BAT lobes were excised from the surrounding WAT and weighed using an analytical balance. BAT was prepared for histological analysis as previously described (62). Primary antibodies used are listed in Supplemental Table 1. Species-matched Cy2-, Cy3-, and Cy5-conjugated IgG secondary antibodies (1:500; Jackson ImmunoResearch Laboratories) were used to detect indirect immunofluorescence. H&E staining was performed as previously reported (61). Slides were imaged with an Olympus IX81 fluorescence or Zeiss LSM710 confocal microscope, with images processed by Zen software (Zeiss). Gross morphology of BAT tissue was imaged with a 16MP camera.

### In vivo metabolic analysis.

Mice underwent a 6 hour fast before i.p. glucose tolerance testing and i.p. insulin tolerance testing were conducted as previously described (62). For cold challenge, basal core body temperature was measured via rodent rectal probe (Microtherma 2; Thermoworks). Mice were individually separated into empty boxes without food, water, or bedding, then placed at 4°C. Core body temperature was measured every hour for 3 hours.

### Body composition and indirect calorimetry.

Lean and fat mass were measured immediately before indirect calorimetry, using noninvasive NMR spectroscopy (EchoMRI; Echo Medical Systems) at the University of Alabama at Birmingham Nutrition Obesity Research Center Small Animal Phenotyping Core. A combined indirect calorimetry system was used to measure EE, feeding behavior, locomotor activity, and fuel use simultaneously (Comprehensive Laboratory Animal Monitoring System; Columbus Instruments) as previously described (63, 64). Oxygen consumption and CO_2_ production were measured every 15 minutes to determine respiratory quotient and EE (65).

### Quantitative real-time PCR.

RNA was isolated from whole tissues using the RNeasy Lipid Mini Kit (Qiagen; catalog 74136). Complementary DNA was synthesized by RT-PCR using Bio-Rad SuperScript III. Single-gene quantitative PCR was performed using iTaq SYBR Green (Bio-Rad; catalog 172-5124) using a CFX96 Real-Time System (Bio-Rad). Data was analyzed using 2^-ΔΔCT^ method. See Supplemental Table 2 for a list of primers.

### Adoptive transfer of diabetic splenocytes.

Islet-transplanted NOD.*Rag* recipients that were euglycemic for 2 weeks were adoptively transferred with 1 × 10^7^ diabetic female NOD splenocytes i.v., as previously described (66). Diabetes was confirmed by glucosuria and two consecutive blood glucose readings ≥ 300 mg/dL, as described previously (61).

### Flow cytometry.

Single-cell suspensions of excised kidney were made as described (46). To process BAT, tissue was sheared into small pieces, incubated with collagenase P (MilliporeSigma) for 30 to 40 minutes at 37°C with periodic shaking, filtered through a 40 μm filter, and then resuspended at 2 × 10^7^ cells/mL. Fc receptors were blocked (BioXCell: BE0307, anti-mouse CD16/CD32), and surface or intracellular flow cytometry was performed with fluorophore-conjugated antibodies (Supplemental Table 3), as we described previously (46). For intracellular staining, cells were fixed and permeabilized with BD Cytofix/Cytoperm Buffer (catalog 554714) or Foxp3/Transcription Factor Staining Buffer Set (eBioscience; catalog 00-5523-00) per manufacturer instructions prior to staining with intracellular antibodies or appropriate isotype controls. Cells were collected by Attune NxT Flow Cytometer (Thermo Fisher Scientific) with 500,000 events per sample and analyzed with FlowJo (version 10.0.8r1) software. Gating strategy is shown in Supplemental Figure 1.

### Statistics.

All statistical analysis was performed using GraphPad Prism, version 8.4. Determination of the difference between mean values and SD for each experimental group was assessed using either unpaired 2-tailed Student’s *t* tests or 1- and 2-way ANOVA. ANOVA was followed by the multiple-comparison Tukey’s post hoc test. Statistical significance was assigned when *P* < 0.05. All experiments were performed at least 3 separate times, and data were obtained from a minimum of triplicate experiments.

### Study approval.

All experiments were approved and conducted in accordance with University of Alabama at Birmingham IACUC–approved mouse guidelines and the NIH’s *Guide for the Care and Use of Laboratory Animals* (National Academies Press, 1978).

## Author contributions

JMB and JDK designed the research studies, conducted experiments, acquired data, analyzed data, and wrote the manuscript. Indirect calorimetry was conducted by MEY, with analysis performed by JDK. MEY, HMT, and CSH designed research studies, analyzed data, and wrote the manuscript. HMT and CSH are the guarantors of this work and, as such, had full access to all the data in the study and take responsibility for the integrity of the data and the accuracy of the data analysis. JDK and JMB are co-first authors, with authorship order determined as follows. For equality and fairness, JDK is mentored by CSH and JMB is mentored by HMT. Since HMT is last, we thought it would be fair to have listed JDK first. This does not diminish the fact that this manuscript was a true 50/50 collaboration and all parties involved are appropriately recognized as first authors and corresponding authors.

## Supplementary Material

Supplemental data

## Version 1 01/11/2022

In-Press Preview

## Version 2 02/22/2022

Electronic publication
