# Supplementary material for: Islet transplantation into brown adipose tissue can delay immune rejection
Source: JCI Insight. 2022 Feb 22;7(4):e152800. doi: 10.1172/jci.insight.152800 (PMC8876467; doi:10.1172/jci.insight.152800)
Supplement: Supplemental data [file jciinsight-7-152800-s253.pdf]

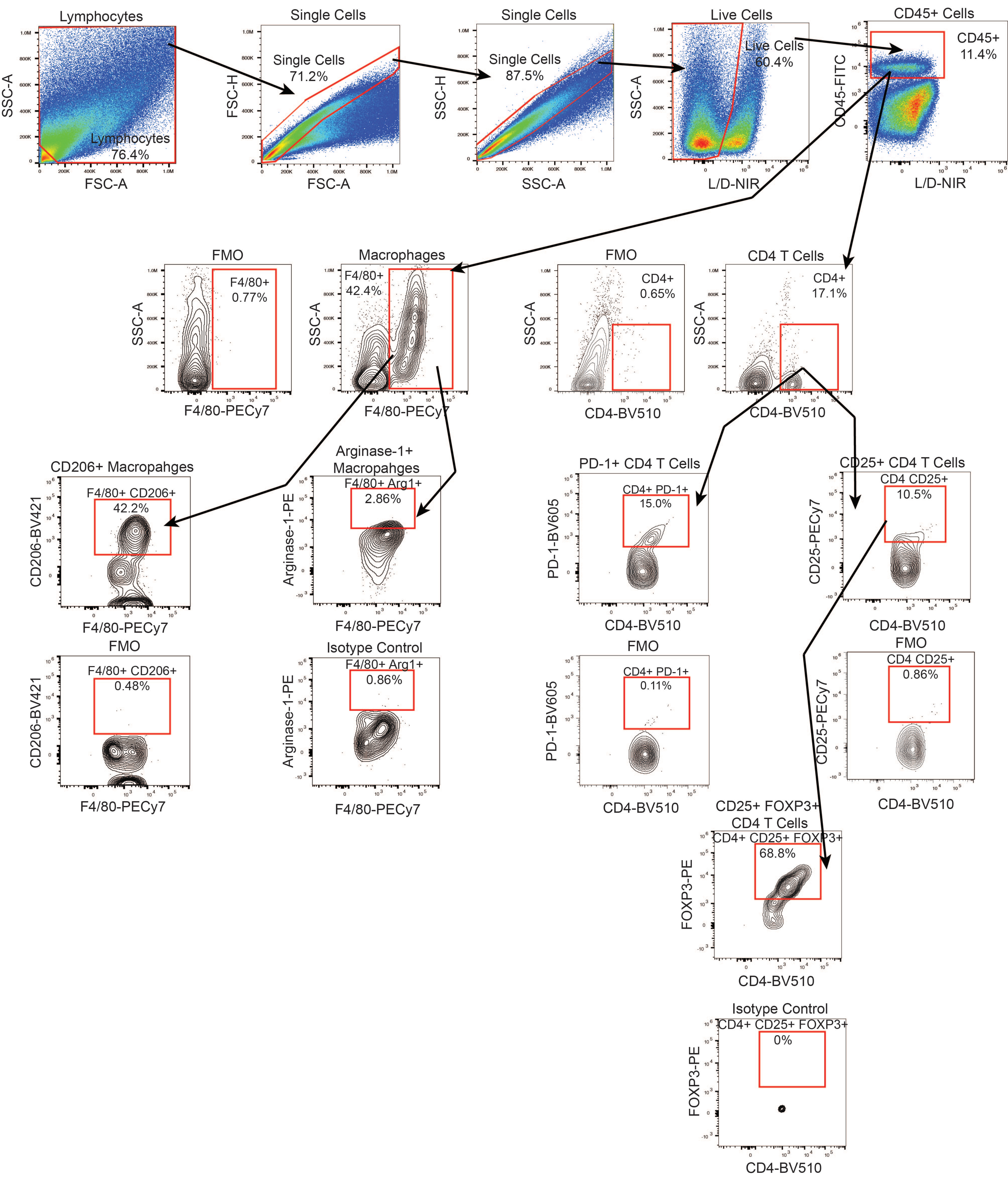

Supplemental Figure 1: Flow gating strategy. Immune cells were pre-gated on single cells, live cells, and CD45+ cells for immunophenotyping of macrophages (F4/80) and CD4 T cells (CD4) before gating on cell surface markers such as CD206 (F4/80+ cells) or CD25 (CD4+ cells). Fluorescence minus one (FMO) for F4/80, CD4, CD206, CD25, and PD-1 and isotype controls for intracellular arginase-1 and FOXP3 are also shown.

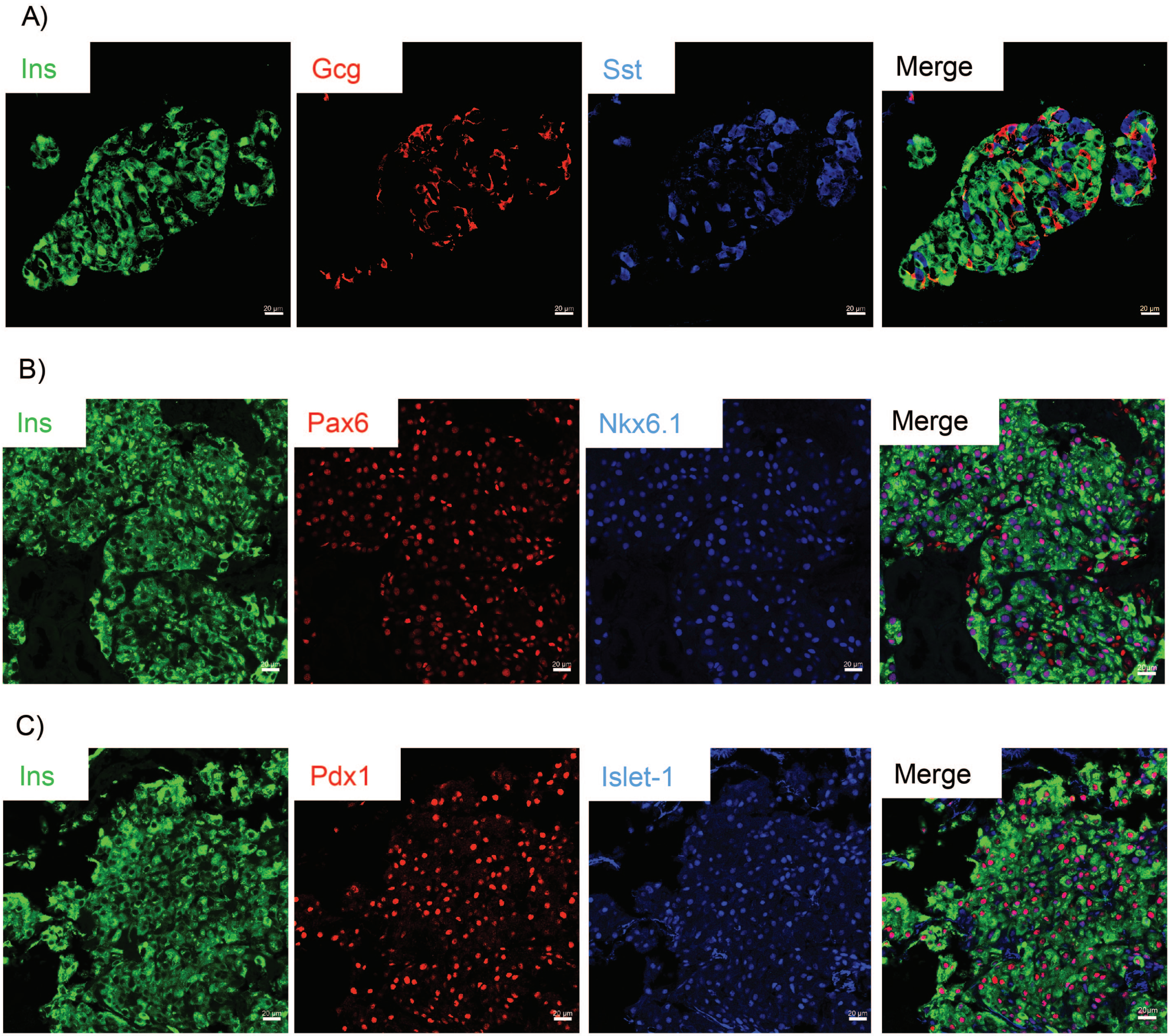

<u>Supplemental Figure 2: Expression of islet hormones and TF in kidney group.</u> Immunofluorescence on kidney transplanted group for A) hormone staining showing insulin (Green), glucagon (Red), and somatostatin (Blue) and B-C) transcription factor staining for Pax6 (Red), Nkx6.1 (Blue) and Pdx1 (Red), Islet-1 (Blue) co-stained with insulin (Green) (n=3).

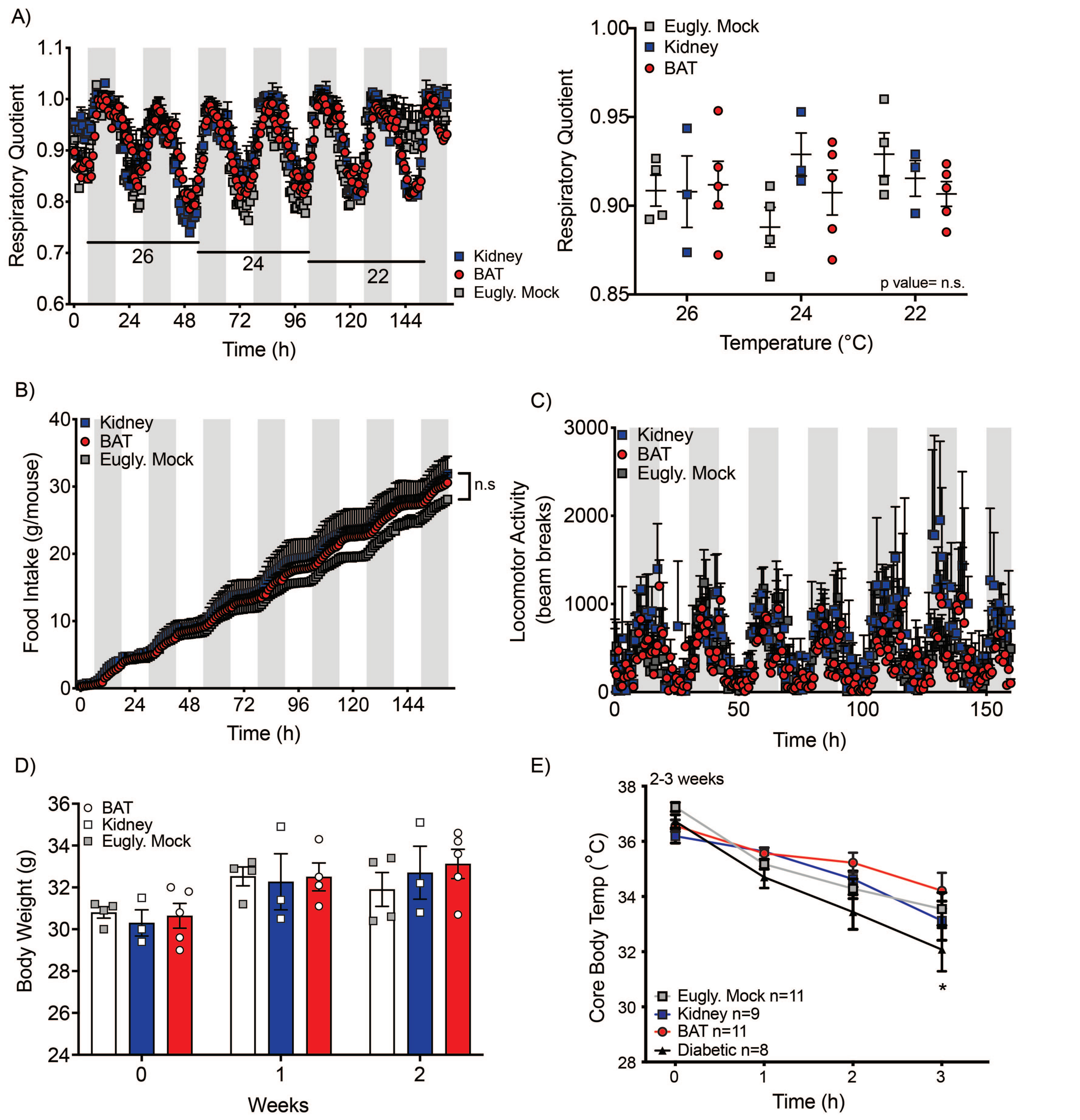

Supplemental Figure 3: Indirect calorimetry analysis. A) respiratory quotient B) food intake and C) locomotion on the kidney transplant group (n=3), BAT (n=5) transplant group, and euglycemic mock (n=4) control as measured with indirect calorimetry. D) Body weight measurements were taken weekly during indirect calorimetry analysis. E) Cold challenge conducted for three hours at 4°C at 2-3 weeks. Energy expenditure analyzed by two-way ANOVA with Tukey post hoc test as compared to euglycemic control. Error bars are +/- SEM. *$p<0.05$.

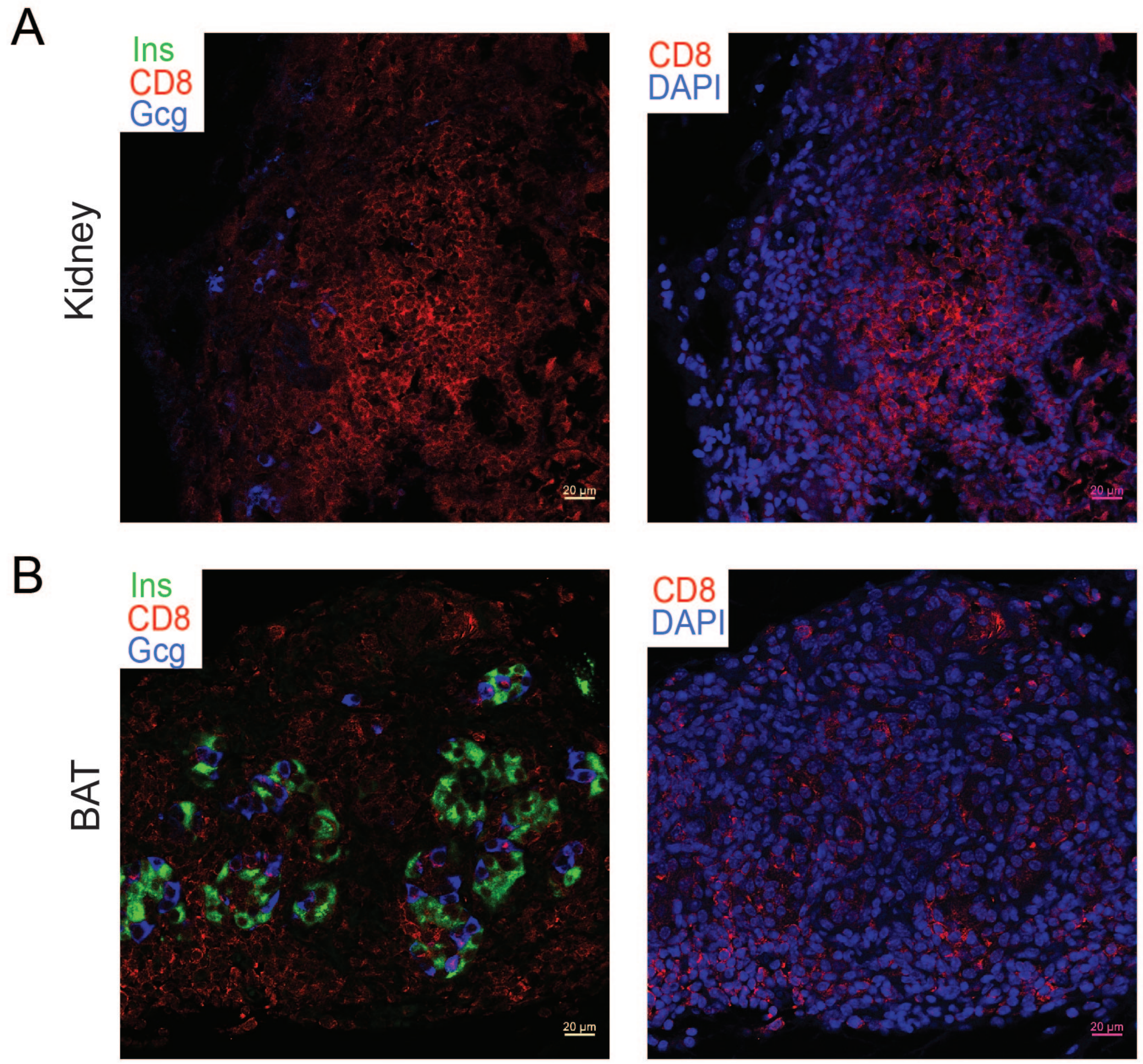

Supplemental Figure 4: Expression of islet hormones and CD8+ T cells in kidney and BAT group. Immunofluorescence on A) engrafted kidney and B) engrafted BAT for hormone staining showing insulin (Green), glucagon or DAPI (Blue), and CD8+ T cells (Red); n=2 for each group.

Supplemental Figure 5

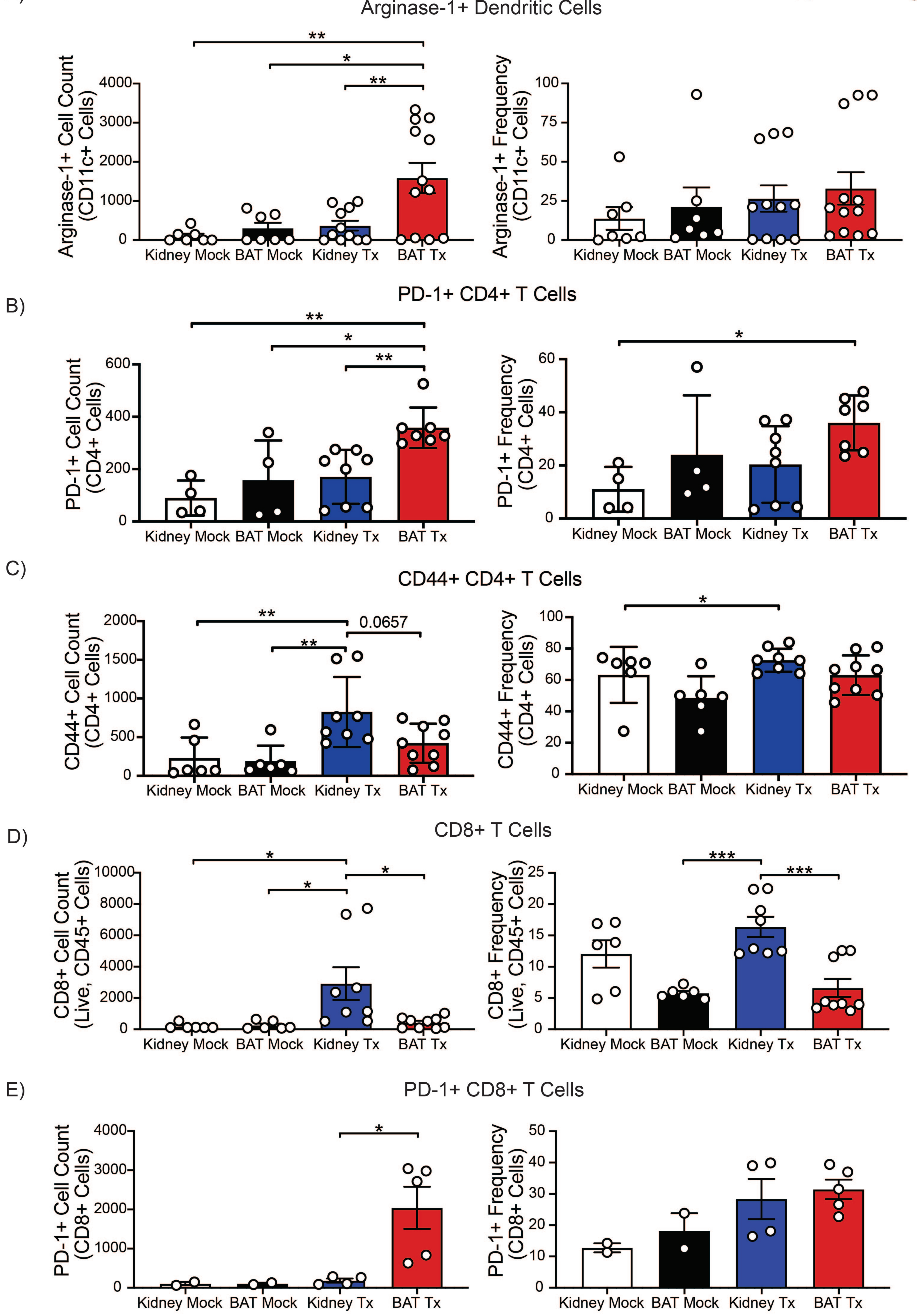

Supplemental Figure 5: BAT has anti-inflammatory immune profile after allogeneic islet transplantation. Flow cytometry analysis of single cell suspensions from allogeneic C57BL/6 islets transplanted into BAT and under the kidney capsule of NOD recipient mice at day 14 post-transplantation. Mock surgery controls are also included for both tissues. Number and frequency of A) arginase-1+ CD11c+ dendritic cells, B) PD-1+ CD4+ T cells, C) CD44+ CD4+ T cells, D) CD8+ T cells, and E) PD-1+ CD8+ T cells (n=2-12). Data were analyzed by one-way ANOVA with multiple comparison and Tukey post hoc test. Error bars are +/- SD; *p<0.05, **p<0.01, ***p<0.001, ****p<0.0001.

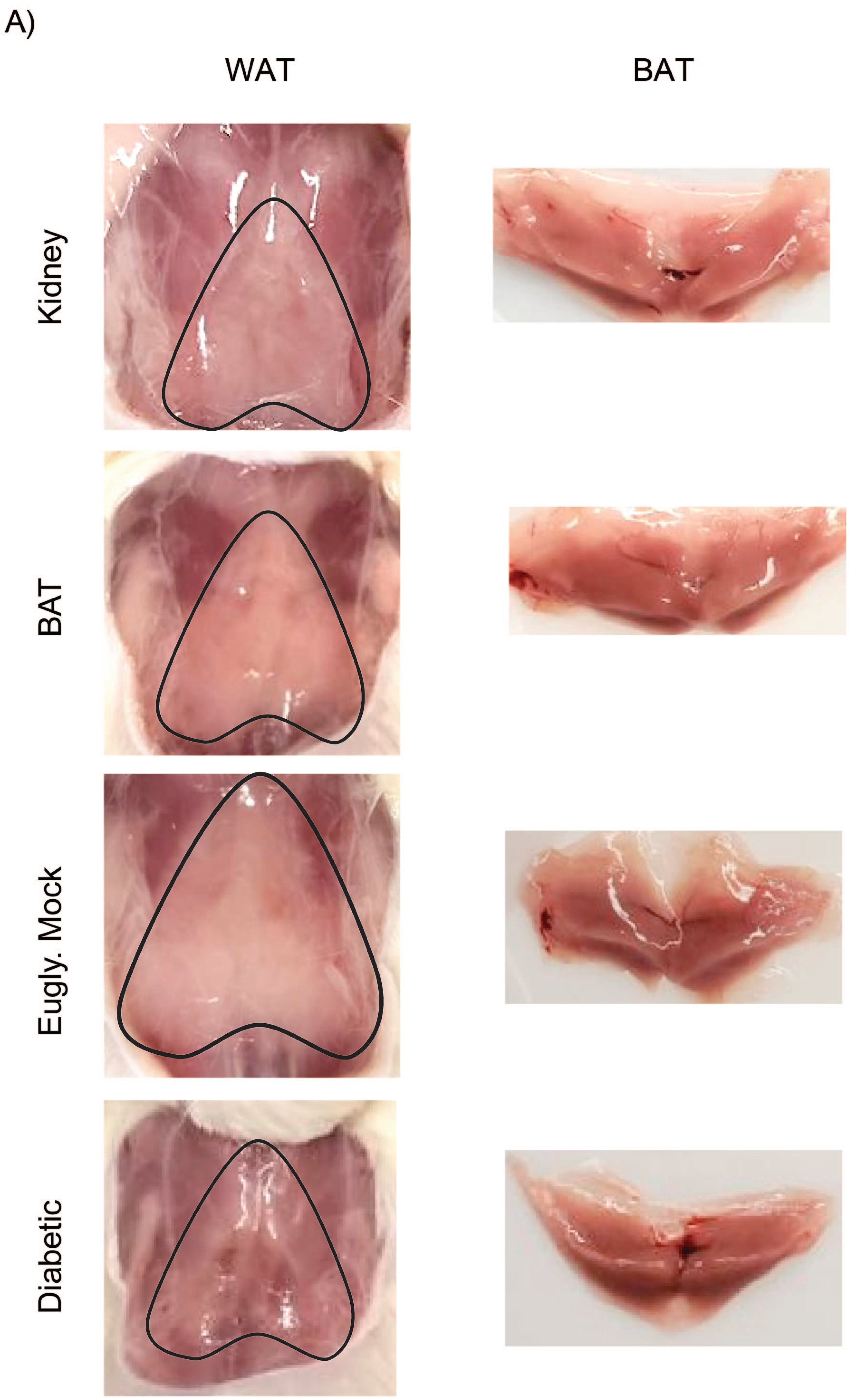

<u>Supplemental Figure 6: Gross morphology of BAT deposit.</u> A) Left image showing dorsal side of white fat pad, outlined in black, covering BAT deposit. Right image showing ventral side of fat pad with two BAT lobes.

Supplemental Materials

Supplemental Table 1: Immunofluorescence Antibodies

Name	Species	Conc.	Company	Catalog #	RRID
Insulin	guinea pig	1:1000	Dako	0564	AB_2617169
Glucagon	mouse	1:1000	Sigma	G2654	AB_259852
Somatostatin	goat	1:1000	Santa Cruz	sc-7819	AB_2302603
Pdx1	rabbit	1:500	Millipore	07-696	AB_417404
Nkx6.1	mouse	1:500	DSHB	F55A10-c	AB_532378
Pax6	rabbit	1:500	BioLegend	901301	AB_2565003
Islet-1	mouse	1:500	DSHB	39.4D5-c	AB_2314683
F4/80	rabbit	1:50	Cell Signaling	70076S	AB_2799771
CD4	rabbit	1:100	Abcam	ab183685	AB_2686917
CD31	rat	1:500	BD Biosciences	553370	AB_394816

Supplementary Table 2: Mouse qPCR Primer Pairs

Gene Name	Forward	Reverse
Ins1	*CCA GCT ATA ATC AGA GAC CA*	*CCA GGTGGG GAC CAC AAA GA*
Ins2	*CCC AGC TCC AGT TGT TCC AC*	*CCA CCC AGG CTT TTG TCA AA*
Gcg	*CAT TCA CCA GCG ACT ACA GCA A*	*TCA TCA ACC ACT GCA CAA AAT CT*
Sst	*AAC GCA AAG CTG GCT GCA AGA A*	*TCA GAG GTC TGG CTA GGA CAA CAA*
Pdx1	*CGG CTG AGC AAG CTA AGG TT*	*TGG AAG AAG CGC TCT CTT TGA*
MafA	*CCT GTA GAG GAA GCC GAG GAA*	*CCT CCC CCA GTC GAG TAT AGC*
MafB	*AAC GCG CCA GCA GAA ACA*	*AGC TGC TCC ACC TGC TGA AT*
Nkx6.1	*CCT CTG GAC CCG AAC TCT GA*	*GCT GCC ACC GCT CGA TT*
Pax6	*TGG CAA ACA ACC TGC CTA TG*	*TGC ACG AGT ATG AGG AGG TCT*
Isl1	*GCA ACC CAA CGA CAA AAC TAA*	*CCA TCA TGT CTC TCC GGA CT*
Ucp1	*GTT CTT GCA CTC ACG CCT CT*	*TGC GAA CCT CAT CAC TCG T*
Zic1	*CAG TAT CCC GCG ATT GGT GT*	*GCG AAC TGG GGT TGA GCT T*
Adrb3	*TCC TTC TAC CTT CCC CTC CTT*	*CGG CTT AGC CAC AAC GAA CAC*
Ppara	*GCG TAC GGC AAT GGC TTT AT*	*GAA CGG CTT CCT CAG GTT CTT*
Pparg	*GTG CCA GTT TCG ATC CGT AGA*	*GGC CAG CAT CGT GTA GAT GA*
Dio2	*CAG TGT GGT GCA CGT CTC CAA TC*	*TGA ACC AAA GTT GAC CAC CAG*
TBP	*GAA GCT GCG GTA CAA TTC CAG*	*CCC CTT GTA CCC TTC ACC AAT*

Supplementary Table 3: Flow Antibodies

Antibody (Dilution)	Clone	Company	RRID
FITC α-CD45 (1:64)	30-F11	BioLegend; 103108	AB_312973
PE α-Arginase-1 (10μL/test)	Polyclonal	R&D Systems; 1C5868P	AB_2889831
PerCPCy5.5 α-CD80 (1:128)	16-10A1	Becton Dickinson; 560526	AB_1727514
PECy7 α-F4/80 (1:64)	BM8	eBioscience; 25-4801-82	AB_469653
BV510 α-CD11c (1:64)	N418	BioLegend; 117353	AB_2686978
AF700 α-CD86 (1:128)	GL1	Becton Dickinson; 560581	AB_1727517
Fixable Live/Dead NearIR (1:150)	N/A	Becton Dickinson; L10119	N/A
BV421 α-CD206 (1:64)	C068C2	BioLegend; 141717	AB_2562232
PE α-Foxp3 (1:16)	FJK-16s	eBioscience; 12-5773-80	AB_465935
BV510 α-CD4 (1:64)	RM4-5	Becton Dickinson; 563106	AB_2687550
BV605 α-PD-1 (1:64)	29F.1A12	BioLegend; 135220	AB_2562616
BV421 α-CD8a (1:32)	53-6.7	BD; 563898	AB_2738474
BV711 α-IFNγ (10μL/test)	XMG1.2	Becton Dickinson; 564336	AB_2738752
BV510 α-CD44 (1:64)	IM7	BD; 563114	AB_2738011
PE α-CTLA-4 (1:64)	UC10-4B9	eBioscience; 12-1522-81	AB_465878
PE α-Sheep IgG Control (10μL/test)	Polyclonal	R&D; IC016P	AB_10645744
PE α-Rat IgG2a Control (1:16)	eBR2a	eBioscience; 12-4321-80	AB_1834380
BV711 α-Rat IgG1 Control (10μL/test)	R3-34	Becton Dickinson; 563283	AB_2869482
